# Birth registration in Nepal: An assessment of progress based on two national surveys

**DOI:** 10.1371/journal.pgph.0000759

**Published:** 2023-01-26

**Authors:** Sharad K. Sharma, Dhruba R. Ghimire, Dadhi Adhikari, Shyam Thapa

**Affiliations:** 1 Office of the Prime Minister and Council of Ministers, Government of Nepal, Kathmandu, Nepal; 2 Asian Demographic Research Institute, Shanghai University, Shanghai, China; 3 South Asian Institute for Policy Analysis and Leadership, Kathmandu, Nepal; 4 Nepal Public Health Foundation, Kathmandu, Nepal; International Institute for Population Sciences, INDIA

## Abstract

Birth registration, an essential component of the civil registration system, is expected to be complete and universal. This study assesses the progress made in recent years and identifies gaps in birth registration in Nepal. Data from the Multiple Indicator Cluster Surveys undertaken in 2014 and 2019 are used for the analysis. The two surveys included a total of 12,007 children under five years of age living with their mothers at the time of the surveys. The survey respondents were 11,821 mothers and 186 caretakers (in the case of those without mothers) of the children. The variations in the proportion of births registered among various subgroups of the children are assessed by performing bivariate analysis and binary logistic regression. Birth registration increased considerably, from 58% (95% CI: 57–59%) in 2014 to 77% (95% CI: 76–78%) in 2019. Several of the disparities between and among the various population subgroups that were evident in the 2014 survey had been considerably reduced or eliminated by 2019. The disparities in registration between boys and girls attenuated over time. Although birth registration increased for all children (ages 0–59 months old), infants still had comparatively lower levels of registration. The relatively disadvantaged provinces showed significant progress between the two survey periods. Considerable and significant progress has been made in birth registration in recent years. However, achieving universal and complete birth registration would require sustaining recent achievements and applying proven strategic interventions to ensure the inclusion of the unregistered births.

## Introduction

Civil registration is defined as the “universal, continuous, permanent and compulsory recording of vital events provided through decree of regulation in accordance with the legal requirement of each country” [1]. Vital events comprise live birth, death (including fetal death), marriage, separation/divorce, and adoption. The four basic principles underlying civil registration include universal coverage, continuity, confidentiality, and regular dissemination. Civil registration is also an important source for tracking most of the Sustainable Development Goal (SDG) 3 targets [2].

As defined, birth registration is an essential component of the civil registration system, and therefore, obtaining a birth certificate is recognized as one of the rights of every child [3]. Despite this global recognition, while considerable emphasis has been placed on promoting access to maternal and child health services, a similar level of commitment has not been accorded to completing birth registration [4]. Globally, 35% of births remain unregistered [5]. Within the South Asia region, as of 2019, Bhutan, Maldives and Sri Lanka had achieved nearly universal coverage of birth registration, while Afghanistan, Pakistan and Bangladesh exhibited lower levels of birth registration coverage than Nepal (77%) and India (86%) [6].

Various factors have been found to account for the variations in the coverage of birth registration across countries. A few studies have examined the determinants of birth registration at the individual, household, and community levels. At the individual level (i.e., mother or parents), those with higher socioeconomic status, higher levels of education, exposure to mass media, higher mobility, and having had an institutional delivery are likely to register their child’s birth [4, 7–10]. A mother’s contact with the health delivery system and/or health care providers have been also found to be facilitating factors across some countries [11]. At the meso and macro levels, the existence of an adequate legislative framework and related infrastructure are also important for achieving a higher level of birth registration [12].

To our knowledge, no such studies have been undertaken for Nepal. As a signatory to the United Nations Economic and Social Commission for Asia and Pacific (UNESCAP) Ministerial Declaration, Nepal is committed to achieving its three goals–attaining universal civil registration; accepting civil registration as legal documentation to claim identity, civil status and ensuring rights to all individuals; and the production and dissemination of timely and complete vital statistics [13]. Based on the insights from previous studies, along with the policy and program interventions that have been undertaken in recent years in Nepal, in this paper we assess the influence of selected factors on the coverage of birth registration, and also identify challenges towards making robust birth registration a part of the overall civil registration system in the country.

Nepal’s 2011 census recorded the total population as 26.5 million, with 595,000 annual births (with an implied crude birth rate of 22.4 per 1,000) [14]. As per the ‘medium variant’ projection, the total population increased, as of 2019, to 29.6 million with 631,000 total annual births. In 2011, only 17% of the total population lived in urban areas, and the percentage was expected to increase to just over 21% by 2019. As of 2016, 53% of currently married women were using a contraception method [15]. The lifetime fertility rate had declined to 2.3 per (woman) based on the averages of three-years (2014–2016) and, furthermore, the average desired number of children had declined to the replacement level– 2.1 per woman of reproductive age–as of 2016 [15]. During the period 2006–2011, only 35% of all deliveries took place at a health facility (institutional delivery) [16]. Five years later, the number of institutional deliveries had increased, accounting for 57.4% of all deliveries–an annual increase of 4.5 percentage points. Thus, the fact that about two out of every five live births occur outside of a health facility presents a challenge to the country’s civil registration system. It is further compounded by the fact that in many communities, social and cultural norms prohibit the mother or child from being visited by outsiders, and also restrict their movement outside the natal home.

## Evolution of the civil registration system in Nepal

The history of registering vital events in Nepal goes back to the 1950s and has been systematically documented by Gautam [17]. He has also pointed out several existing challenges and barriers, including legal complexities and ambiguities, lack of proper documentation, and discrimination against poor and vulnerable groups, among others.

The current (2015) Constitution of Nepal recognizes birth registration as a right for every child, and has given authority to all three levels of the government–federal, provincial, and local–for registration of vital events [18]. Accordingly, all municipalities and other designated places are mobilized for the registration of vital events. In order to facilitate the civil registration process, the Government established the Department of Civil Registration in 2015. The National Identity Card and Registration Act of 2020 and the National Identity Card, along with the Registration Regulation of 2021, are also part of the civil registration system [19–21]. The Department of National ID and Civil Registration (DoNICR) is responsible for both registration of vital events and identity management [22]. Accordingly, an electronic registration database has been developed and birth registration has also been made mandatory for school admission [22].

As of 2020, a total of 6,743 local registrars have been deployed in 753 municipalities for registration and issuance of birth certificates [22]. The local offices can also take punitive action for registration related delays and fraud. Birth registration has to be performed by the local ward office within 35 days of delivery, and requires the signature and citizenship of parents [23]. The completed birth registration forms are entered in the birth registration ledger. The local ward secretary issues the birth certificate and the chairperson of the ward signs and stamps the birth certificate.

Aside from the structural and organizational set up, some significant policy and program interventions have been launched that have bearing on the program’s effectiveness and improvements in birth registration. Beginning in 2009, the Government of Nepal introduced a cash transfer incentive program to parents to register their children, particularly in comparatively socioeconomically disadvantaged provinces [24]. Additionally, the government introduced a nationwide program to encourage the participation of conventionally disadvantaged groups–targeting Dalit communities in particular. These specific policies and programs have been noted to have had positive impacts [25–27]. Similarly, the government has introduced various programs to raise community awareness regarding the importance of birth registration, including avoidance of discrimination against the girl child in birth registration, among many other community- and province-based programs [28].

These steps seem to have paved the way for steady progress in recent years. Nationally, birth registration increased from 35% in 2006, to 42% in 2011, to 53% in 2014, to 56% in 2016, and to 77% in 2019 [15, 16, 29–31]. Thus, between 2011 and 2019, there has been an increase of 35 percentage points in birth registration. Still, these national coverage rates conceal variations that may exist between and among the population sub-groups and geographical areas. Identifying these disparities could help generate interventions targeted to specific places and population subgroups. The country-specific evidence could also contribute to the global knowledge base that is linked to the SDG 3 targets.

## Data and methods

The data analyzed are from the Nepal Multiple Indicator Cluster Surveys (NMICS) undertaken in 2014 and 2019. The NMICS are standardized cross-sectional and nationally representative household surveys conducted by the government’s Central Bureau of Statistics (CBS) in partnership with UNICEF/Nepal [30, 31]. The 2014 and 2029 surveys included 13,000 and 12,800 households, respectively.

The surveys ascertained the birth registration status of each child under five years of age living with a mother or caretaker in the household on the day of the interview. In the 2014 survey, there were a total of 5,279 mothers and 70 caretakers (non-biological female caretakers). Similarly, in the 2019 survey, there were 6,542 mothers and 116 caretakers. They were the respondents in the survey who provided information relating to the birth registration of children– 5,349 in the 2014 survey and 6,658 in 2019 survey. These under-five living children are defined as the ‘study population’ for this analysis. In view of the fact that the caretakers represent only a small proportion (1.3% in the 2014 survey and 1.7% in the 2019 survey), we refer to all the respondents as ‘mothers’ for convenience.

The outcome variable is the registration/non-registration status of each study population. A child is considered registered as per the verbal reporting–yes or no–of the respondent, regardless of whether the child had a birth certificate or not. The individual-level variables used as the explanatory variables are: age of the child (0–59 months), sex of the child, and the mother’s highest level of education. The household-level variable is represented by the wealth (household) index. The index includes household amenities, assets and durables that are grouped into five quintiles, from the poorest to the richest [32].

Nepal is known to have many ethnic groups intertwined in religious affiliation, specific geographic location, traditional caste, tribal practices, and other considerations [33, 34]. For this analysis, we classified household ethnicity into four broad groups: Brahmin and Chhetri (including Thakuri), Janajati, Dalit, and all other. The first group is generally considered to be the most advantaged group, Janajati refers to the indigenous group, and Dalit are regarded as one of the most disadvantaged ethnic groups. The ‘other’ groups also include Muslim ethic-religious groups. The contextual-level variable is represented by the province in which the respondent (or the child) lives. Of the total of seven provinces, Province 3, where the federal capital lies, is comparatively the most advantaged in terms of multiple indices of socio-economic development, and Provinces 6 and 7 are relatively worse-off [35, 36].

Aside from the six independent factors (variables), additional variables of interest for inclusion could be urban-rural place of residence, mass media exposure, mother’s age, parity, and birth order. In a preliminary analysis, urban-rural place of residence did not show any significant difference. This was probably due to the fact that in recent years many areas that are principally rural in characteristics have been designated as urban areas for political and budget allocation purposes. For example, the 2014 survey reported that 58% of the respondents were living in an urban area, and that number increased to 65% in 2019. (For a comparative perspective, only 35% of the population lived in urban areas in India as of mid-2020, and it is rather difficult to accept that Nepal is substantially more urbanized than India.) As regards the mass media variable, the composite index variable ‘wealth status’ includes the household’s possession of radio and television. Because of the low fertility level, as noted earlier, we excluded birth order or number of live births variables from the analysis. In a preliminary analysis, we also included the mother’s age as an additional variable (by excluding the non-biological female caretakers in the sample). The inclusion of this variable did not make any significant contribution to the overall results. This variable is, therefore, not included in the results presented in the paper. We adapted the strategy of parsimony in the data analysis and modeling. Also, more importantly, the study is focused on testing how certain interventions may have influenced the higher level of registration, and also, evaluating how some of the factors identified here influenced the level of registration.

To the extent that the recent policy and program interventions (mentioned earlier) have contributed to the improvement in birth registration in recent years, we expected to find a strong significant influence on the participation of the Dalit ethnic group in the program in particular, especially those living in the most disadvantaged provinces in the country. Further, the effect of the ‘child grant’ intervention program is expected to be evident in the birth registration based on the socio-economic class of the household where the child resides. Additionally, the child’s age is expected to be strongly and significantly associated with birth registration. More specifically, because a birth registration card is required for children for admission to school, a higher level of uptake birth registration is expected around age five.

As noted earlier, the fairly high level of birth registration– 77% as of 2019 –should necessarily encompass the registration of diverse population subgroups, and not be limited to certain more affluent population subgroups. A similar pattern has been found with respect to the vitamin A supplementation provided to children in ages 6–59 months, when the percentage of children receiving the supplementation progressed to a higher level [37]. In view of the introduction of the intervention programs and the increased level of overall birth registration, we expected to find the disparities between the study population subgroups reduced between the two survey time periods.

Bivariate analysis is performed to examine the association between the outcome variable and the background characteristics. Binary logistic regression is applied to assess the net effect of each of the background variables (covariates) on the outcome variable [38]. The logistic regression model can be described as follows:

Logit (P) = log (P/1−P) = β_0_ + β_1_X_1_ + … + β_k_X_k_, which states that the (natural) logarithm of the odds of outcome variable is a linear function of the *X* variables (covariates), and is often called the log odds. This is also referred to as the logit transformation of the estimated probability of outcome variable, P. The outcome variable is binary: ‘1’ if the index child has had a birth registration, and ‘0’ if no. We considered a *p*-value of 0.05 or better to indicate a statistically significant association for interpretation and drawing inferences and conclusions based on the results.

## Results

The percent distribution of the variables included in the analysis for the two surveys are presented in Table 1. For most of the variables, there are only small differences in percentage between the two surveys, and the small differences are most likely due to the sampling variability. For convenience, we have pooled the data from the two surveys, and the distributions are also shown in the table.

**Table 1:** Distribution of the study sample (all children younger than five years of age living with their mothers at the survey time), 2014 and 2019 surveys, by background characteristics, Nepal.

	2014	2019	2014 & 2019
Characteristics	%	%	%
Age (months)			
0–11	18.3	19.6	19.0
12–24	18.8	19.0	18.9
24–35	20.2	18.5	19.3
36–47	21.3	21.8	21.5
48–59	21.5	21.2	21.3
Sex			
Male	51.7	52.6	52.2
Female	48.3	47.4	47.8
Mother’s education			
None	42.4	25.8	33.2
Grades 1–8	17.2	31.8	25.3
Higher than grade 8	40.4	42.4	41.5
Mother’s ethnicity			
Brahmin/Chhetri	29.2	27.2	28.1
Janjati	30.3	33.1	31.8
Dalit	15.8	15.7	15.7
All other	24.7	24.0	24.4
Household wealth quintile			
1—Poorest	22.1	23.3	22.8
2—Poor	20.3	20.5	20.4
3—Middle	22.0	20.2	21.0
4—Rich	20.3	19.5	19.9
5—Richest	15.3	16.5	16.0
Province of residence			
1	15.3	15.8	15.6
2	25.2	23.7	24.4
3	15.0	18.8	17.1
4	9.1	7.2	8.0
5	18.6	18.2	18.4
6	7.4	6.7	7.0
7	9.5	9.7	9.6
Total	100.0	100.0	100.0
No. of cases	5,349	6,658	12,007

Note: The total for each variable equals 100 unless affected by rounding.

Of the total of 12,007 children included in the two surveys, about one-fifth belong to each of the five age groups. There are more male than female children, with a male-to-female ratio of 110 (per 100). Unlike other characteristics, the two surveys recorded large differences in the proportion of the children’s mothers based on the education categories: whereas the 2014 survey reported 42% of the mothers having no education, it was only 26% in the 2019 survey. Similarly, the 2014 survey recorded 17% of the mothers as having had a basic education (1–8 grades), while it was 32% in the 2019 survey. [However, such a large change over a five-year period is unlikely; it is possible that some of the women who were ‘literate but with no formal years of schooling’ may have been misclassified]. Slightly over one-fourth of the respondents belonged to the Brahmin/Chhetri group, about one-third of the women belonged to the Janajati group, and 16% were in the Dalit group. Nearly one-fourth of the women belonged to the poorest household wealth group, and 16% belonged to the richest group.

Regarding the place of residence, Province 2 had the largest proportion (24%) of the children, while Provinces 4, 6 and 7 had the smallest proportion (7–10%). Province 3, where the federal capital is located, had 17% of the total children residing at the time of the survey. Province 3 also showed a slight increase in the proportion between the two surveys.

Table 2 presents the registration status of the children between the two time periods. As of 2014, 58% (95% CI = 57–59%) of births had been registered, and the percentage increased to 77% (95% CI = 76–78%) over the next five years, indicating an increase of 3.8 percentage points annually. There were, however, considerable variations based on the background characteristics.

**Table 2:** Percentage (with 95% confidence interval) of children registered among all children younger than five years of age living with their mothers at the survey time, 2014 and 2019 surveys, by selected background characteristics, Nepal.

Characteristic	2014		2019		Percent	
	Percent	95% CI	Percent	95% CI	Diff^§^	Change^ǂ^
Age (months)	*p* = 0.00		*p* = 0.00 0.000			
0–11	32.8	29.9–35.8	59.5	56.8–62.1	26.7	81.4
12–23	47.8	44.7–50.8	74.6	72.2–77.0	26.8	56.1
24–35	63.9	61.0–66.7	79.0	76.6–81.2	15.1	23.6
36–47	67.9	65.1–70.5	84.2	82.2–86.0	16.3	24.0
48–59	73.8	71.1–76.2	87.2	85.4–88.9	13.4	18.2
Sex	*p* = 0.15		*p* = 0.13 0.000			
Male	59.2	57.3–61.0	76.3	74.9–77.7	17.1	28.9
Female	57.0	55.1–58.9	78.3	76.8–79.7	21.3	37.4
Mother’s education	*p* = 0.84		*p* = 0.13 0.000			
None	58.3	56.3–60.3	75.5	73.5–77.5	17.2	29.5
Grades 1–8	60.1	56.9–63.2	77.8	76.0–79.5	17.7	29.5
Higher than 8 grade	57.1	55.0–59.1	77.8	76.2–79.3	20.7	36.3
Mother’s ethnicity	*p* = 0.00		*p* = 0.00 0.000	0.000		
Brahmin/Chhetri	54.2	51.8–56.7	80.3	78.4–82.1	26.1	48.2
Janjati	52.5	50.0–54.9	73.4	71.5–75.2	20.9	39.8
Dalit	75.4	72.3–78.2	84.8	82.5–86.9	9.4	12.5
All other	58.6	55.9–61.2	74.1	71.9–76.2	15.5	26.5
Household wealth quintile	*p* = 0.00		*p* = 0.060			
1 –Poorest	54.6	51.8–57.4	79.9	77.8–81.8	25.3	46.3
2 –Poor	58.1	55.2–61.0	74.1	71.7–76.4	16.0	27.5
3 –Middle	62.0	59.2–64.7	78.9	76.6–81.0	16.9	27.3
4 –Rich	58.3	55.3–61.2	78.3	75.9–80.0	20.0	34.3
5 –Richest	57.5	54.0–60.8	74.1	71.4–76.6	16.6	28.9
Province	*p* = 0.00		*p* = 0.00 0.000	0.000		
1	65.7	62.4–68.9	78.4	75.8–80.8	12.7	19.3
2	58.1	55.4–60.7	76.1	73.9–78.1	18.0	31.0
3	44.1	40.7–47.5	70.8	68.2–73.3	26.7	60.5
4	57.3	52.8–61.6	73.8	69.7–77.6	16.5	28.8
5	64.4	61.4–67.4	76.7	74.3–79.0	12.3	19.1
6	75.4	70.9–79.4	84.4	80.7–87.5	9.0	11.9
7	43.0	38.8–47.4	89.1	86.5–91.3	46.1	107.2
Overall	58.1	56.8–59.4	77.2	76.2–78.2	19.1	32.9

^§^absolute difference in percentage values (2014–2019). ^ǂ^ Percent change between the two time periods.

Note: *p*-value is based on bivariate χ^2^ test with two categories–registered and non-registered births–for each variable shown in the table for 2014 and 2019 separately. *p*-value of 0.00 indicates 0.0001.

Compared to the other age groups, the registration of infants in particular (i.e., under one year of age) increased sharply from 33% to 59% between the two time periods. Still, the absolute proportion registered among infants was proportionately less than other age groups. The registration of female children in particular increased proportionately over time, and by 2019, the difference in birth registration between the boys and girls had attenuated.

The increase in the birth registration of children was higher than the overall average, particularly among women with higher levels of education. Among the five broad ethnic groups, as of 2019, the Dalit had the highest proportion (85%) of children registered; even higher than among the Brahmin/Chhetri group. The poorest household group had proportionately the highest gain (46%) in birth registration, while the richest group experienced only a modest gain (29%). But the coverage across all the five groups remained high—between 74% and 80%.

Two of the seven provinces experienced a considerable gain in registration over time. Province 7 showed the largest gain (107%) followed by Province 3 (61%). Province 6 showed the smallest gain (12%). This was, however, also due to an already high level of coverage in the base year (2014). Overall, all of the provinces except for Province 3 had reached coverage levels between 74% and 89% as of 2019.

We performed a multivariate binary logistic regression to assess the net effects of the six covariates (variables) on birth registration. The results (Table 3) indicate a considerable change between the two time periods. First and foremost, whereas large and significant differentials existed as of the 2014 time period, most of the differentials subsided by 2019. Even when the differentials persisted, the effects were generally much smaller than during the earlier period.

**Table 3:** Odds ratio (OR) of the covariates associated with the registration of children among all children living with their mothers at the time of the survey, 2014 and 2019 surveys, multivariate binary logistic regression results, Nepal.

	2014		2019	
Covariates	OR	95% CI	OR	95% CI
Age (months)				
0–11	1.0		1.0	
12–24	2.1**	1.7–2.6	1.9**	1.6–2.3
24–35	3.9**	3.2–4.8	2.6**	2.2–3.1
36–47	4.8**	3.9–5.8	3.9**	3.2–4.8
48–59	6.2**	5.0–7.7	5.3**	4.3–6.6
Sex				
Male	1.0		1.0	
Female	0.9*	0.8–1.0	1.1	1.0–1.2
Mother’s education				
None	1.0		1.0	
Grades 1–8	1.2	1.0–1.4	1.1	0.9–1.4
Higher than 8 grade	1.3*	1.0–1.5	1.4**	1.1–1.7
Mother’s ethnicity				
Brahmin/Chhetri	1.0		1.0	
Janjati	0.9	0.7–1.2	0.9	0.8–1.1
Dalit	2.8**	2.1–3.8	1.8**	1.4–2.4
All other	1.0	0.7–1.4	0.8	0.6–1.0
Household wealth quintile				
1—Poorest	1.0		1.0	
2—Poor	1.4**	1.1–1.7	1.1	0.8–1.4
3—Middle	2.0**	1.5–2.7	1.4*	1.0–1.8
4—Rich	1.8**	1.3–2.4	1.2	0.9–1.6
5—Richest	1.9**	1.4–2.6	1.1	0.8–1.5
Province				
1	2.1**	1.5–2.8	1.2	0.9–1.6
2	1.4	1.0–2.0	1.1	0.8–1.6
3	1.0		1.0	
4	1.7**	1.1–2.5	0.9	0.7–1.2
5	2.2**	1.6–3.1	1.2	0.9–1.6
6	7.5**	4.5–12.7	1.9**	1.2–2.8
7	1.1	0.8–1.5	2.9**	2.0–4.3
Log likelihood	-3,166		-3,134	
No. of cases	5,349		6,658	

*p<0.05

**p<0.01; OR = odds ratio; CI = confidence interval

In both the survey periods, birth registration increased consistently for all children in ages 0 to 59 months. However, compared to the children more than 12-months old, infants had persistently lower odds of being registered. The lower registration of female children that existed in 2014 had also subsided by 2019, indicating that the sex disparity in birth registration attenuated considerably over time.

Educated mothers still have a slightly higher advantage in having their child’s births registered compared to their counterparts with less education. Among the ethnic groups, Dalit showed higher odds of having their births registered (compared to Brahmin and Chhetri). The poorest households did just as well as the other households. More importantly, the large differentials and disparities in birth registration that existed across the provinces as of 2014 had been minimized by 2019. A similar pattern of change occurred across all the provinces of residence. Provinces 6 and 7, known to be relatively the most disadvantaged in terms of socio-economic indicators, actually experienced a significant likelihood of having under-five children registered (OR of 2.9, compared to the reference province, Province 3).

### Discussion

Higher birth registration, particularly among the Dalit (officially designated as an oppressed and deprived ethnic group), and in comparatively worse-off provinces, seems to be the result of specifically targeted policy and program interventions [30, 39]. Beginning in 2009, the government introduced a ‘child grant’ program targeted to children under the age of five, particularly in Province 6, that was expanded to Provinces 2 and 7 over the years [24, 40]. Similarly, a special national program targeted toward the Dalit community was also launched so that their participation in the birth registration program could be strengthened.

The ‘child grant’ program comprised a cash transfer to the child’s family to inform, empower, and encourage them toward the birth registration of the child. This program most likely affected the household economic situation, particularly for the poorest families, as was also evident from the 2019 survey results. The impact of the cash transfer intervention program ─ in the form of child grant or social security programs ─ has been documented in several other countries as well [25–27, 41–44], and Nepal’s experience conforms to this globally emerging evidence base.

The data indicated that the proportion of children registered increased rapidly with the child’s age, and reached nearly 90% by age five. This pattern is most likely linked to the school-going time of the child, as a birth certificate is required for admission to school. The age-related pattern of birth registration has also been found in other countries [45–47]. This also suggests that children who are not enrolled in school may still be left out of the registration process. Unlike in the past, the proportion of children not enrolled in school is low, but this non-enrollment still affects a small proportion of children, particularly female children [48, 49]. The results also point to the need to identify social and culturally appropriate strategies for reaching out to the parents of younger aged children in particular to birth register their children.

As noted in the preceding section, the data on the mother’s education between the two time periods are difficult to explain (particularly, the proportion of mothers with ‘no education’ and 1–8 grades education). We know of no specific interventions targeting the education of the mothers. However, it is possible that some of the women who were ‘literate but had no formal education’ may have been misclassified. Notwithstanding this, the results showing that the mothers with a higher than 8^th^ grade education are more likely register their children may indicate a higher level of awareness, realization of the importance of birth registration, and self-efficacy.

The data also clearly indicate that the typical biases against girls that are known to prevail in patrilocal and patriarchal societies such as in Nepal are not as pronounced as they might have been in the past. As a matter of fact, with respect to their birth registration, the differences between boys and girls had diminished by 2019. Similar findings also existed with respect to vitamin A supplementation for under-five children in Nepal [37]. Part of this newly emerging reason could be that there has been a rapid decline in fertility over recent time periods in Nepal. Lifetime fertility has declined to 2.3 per woman, with only a slightly higher number for rural areas [15]. In societies where preference for a particular sex of children is not a social or cultural norm, having at least one child (regardless of the sex) is the overriding consideration; at the other extreme, in families where there is a preference for a large number of children, the probability of having at least one male child is high [50]. In the case of Nepal, where son preference has remained prevalent for time innumerable, a new reproductive norm may be emerging where having or not having a child may be a more important factor than having a child of a particular sex. The larger social, cultural and attitudinal milieu may be changing to a more cosmopolitan norm. However, this remains speculative. But, the fact that the differential in mother’s behavior based on the sex of the child with respect to birth registration (and also with respect to the vitamin A supplementation) are most likely indicative of changing practices.

Further, underpinning the evidence of progress indicated by the individual level data is the fact that the basic organizational infrastructure warranted for a functioning civil registration system that necessarily includes birth registration is gaining a foothold in the country. In this context, the Department of National Identity and Civil Registration (DoNICR) appears to be functioning increasingly effectively. Policies and programs initiated by the DoNICR include building the capacity of local government bodies, raising awareness, addressing perceived or actual barriers to registration, and other related activities towards achieving complete and universal birth registration have been launched with periodic supplementary activities [22, 27, 40]. The importance of this meso and macro level implementation of policies and programs needs to be taken into account in the context of understanding the necessary components for sustained progress over time.

## Limitation

A major limitation of the data analyzed here is that no birth registration information was ascertained for children who may have died among the live born during the five years immediately preceding the survey time. Based on the under-five mortality estimates from the same surveys, this implies missing information on 3.8% and 2.8% of live births in the 2014 and 2019 surveys [30, 31]. However, even if the survey had attempted to ascertain this registration information, it is likely that the information would not have been complete, especially if a child died during infancy. Furthermore, the mothers may not have wished to share this information. Second, because the surveys were *de facto* surveys (that is, they included only the children who were in the household at the time of survey), it excluded those who may have been away or traveling at the time of the survey, although this is likely to be a small proportion. Third, all the births that were reported and considered as registered in the surveys may not necessarily have had a birth certificate. Finally, the survey data analyzed do not include several factors such as distance, convenience and ease of registration, availability of trained and skilled human resources, and access to healthcare contacts. These factors have been found to be important enabling or restricting factors associated with higher coverage of birth registration [4, 7, 8, 11]. Additional factors may include the attitudes and perceptions towards birth registration held by parents and communities. The influence of these factors could be explored through studies focused on birth registration, and may also warrant a mixed methodology in terms of data collection and analysis. By way of improving upon the data analysis (modeling), a multilevel analysis (e.g., women respondents are nested within the clusters, clusters nested within the districts, and districts nested within the provinces) could help to strengthen our understanding of the influence of meso and macro levels affecting the birth registration [4, 45].

## Conclusion

The analysis of the data on birth registration, based on the surveys from the two time periods– 2014 and 2019 –unequivocally indicate that there have been significant gains in the registration of births of children under five years of age immediately preceding each survey. Thus, the two surveys provide a snapshot of an approximately 10-year period. As of 2019, more than three-fourths (77%) of births are registered, and this should be considered a remarkable achievement. This record of achievement has placed Nepal ahead of some other countries within the South Asia region.

The data also clearly indicate that the typical differences between boys and girls are no longer prevalent as regards birth registration. Similarly, the other large disparities based on household wealth status have been considerably minimized. At the same time, some other disparities remain and need attention. A large proportion of infants remain unregistered, and this warrants socio-culturally appropriate interventions. The mothers with little or no education also need attention. Additionally, respondents living in better-off geographical areas (that is, comparatively better-off provinces) actually have lower birth registration rates than those living in relatively more disadvantaged areas. On the other hand, the evidence from relatively disadvantaged provinces indicates that it is feasible to increase the coverage if focused interventions are initiated. To this end, the evidence analyzed in this paper could provide guidance towards understanding where the disparities exist so that targeted interventions could be designed, implemented, and evaluated.
